# A Review on Ophthalmic Manifestations of Brucellosis and Reporting a Case of Ophthalmic Brucellosis

**Published:** 2011-05-01

**Authors:** R Ghasemi Barghi, H Meraat, A A Pahlevan

**Affiliations:** 1 Department of Infectious Disease, School of Medicine, Qazvin University of Medical Sciences, Qazvin, Iran; 2 Department of Ophthalmology, School of Medicine, Qazvin University of Medical Sciences, Qazvin, Iran; 3 3Department of Microbiology, School of Medicine, Qazvin University of Medical Sciences, Qazvin, Iran

**Keywords:** Brucellosis, Uveitis

Dear Editor,

Considering the severe outcome of undiagnosed ophthalmic brucellosis, the timely recognition of this form of disease could prevent its further complications.[1] The first case of ophthalmic brucellosis was reported by Lemaire in 1924.[2] The eye involvement in brucellosis occurs in different forms including dacryoadenitis, conjunctivitis, episcleritis, keratitis, iritis, iridocyclitis, neuroretinitis, retinitis, chorioiditis, panuveitis, pars planitis, and hyalitis. The clinical manifestations of ophthalmic brucellosis include injection, blurred vision, eye pain, tearing, diplopia, foreign body sensation, cotton-wool lesions, exudative retinal detachment, and retinal hemorrhage.[1][2][3][4][5] There are two explanations towards the pathogenesis of ophthalmic brucellosis including direct presence of brucellae and immune complexes depended pathogenesis.[2][4][6]

Rolando et al. (2008) described two different types eye involvement compromising ophthalmologic and neurophthalmologic ones.[2] Puig Solanes et al. (1953) described that among 60 patients with ophthalmic complication of brucellosis, 48 were with neurophthalmic involvement.[3] In majority of studies, uveitis has been considered as the most frequent form of ophthalmic involvement and posterior uveitis was the most common form of uveitis.[2][3][6][7] However, there is only one report in which conjunctivitis is shown to be the most common clinical form of ophthalmic brucellosis.[8]

The appearance of eye involvement in most cases of disease occurs during the chronic phase of brucellosis.[2][3][8] Our patient also developed uveitis during the chronic phase of brucellosis, a finding consistent with data of other studies. This is the first report of ophthalmic brucellosis in Qazvin Province as a bilateral involvement. The patient was a 45-year old man with fever, chills, and anorexia lasting for the last three months. He gave the history of consuming unpasteurized milk and cheese. Slight blurred vision of ten days duration was present at the time of the first medical appointment. The vital signs were stable. Fever, backache and arthralgia were the only main complications. The results of primary laboratory tests were SAT: 1/320, and Coomb’s Wright: 1/640.

Anti-brucellosis therapy was initiated by administration of doxycycline (100 mg/12 hr) and rifampin (300 mg/12 hr). A week later, the patient returned because of the severity of blurred vision and floater. On ophthalmologic examination, posterior uveitis as choroiditis, signs of vitritis and the presence of inflammatory cells within the vitrea at a range of 1(+) were reported while the retina, conjunctivae, optic disk, and intra-ocular pressure were described as normal. The vision of both eyes following its best correction was at a limit of 8/10. The following tests were requested and the results reported were negative for PPD, VDRL, FTAABS, RF, ANA, and anti-toxoplasma Ab (IgM, IgG) as well as normal chest X ray (CXR).

Considering the positive serologic tests for brucellosis and rejection of other causes of uveitis, the patient was diagnosed as an ophthalmic brucellosis and therefore, in addition to anti-brucellosis therapy, prednisolone at a daily dose of 50 mg was also administered. After 10 days, the patient’s vision improved considerably and after day 14, prednisolone dose was tapered and discontinued within 3 weeks while anti-brucellosis treatment continued for 8 weeks. Ophthalmologic re-examination a month following recovery was indicative of significant improvement of uveitis. The patient’s vision increased to 10/10. At the end of treatment, the patient was free of any clinical signs and symptoms, also with no signs of recurrent brucellosis and eye involvement after one-year follow-up.

The most common symptom of ophthalmic brucellosis was blurred vision that in most cases occurs bilaterally. In a study by Rolando et al., it was demonstrated that among 52 patients with signs of ophthalmic brucellosis, 69.2% were with blurred vision. Interestingly, 19.3% of patients with no obvious ophthalmic symptoms had abnormal sight and pathologic findings when subjected to ophthalmologic examinations.[2]

Diagnosis of ophthalmic brucellosis is made through clinical ophthalmic examinations and laboratory tests including standard agglutination tests (SAT), Coomb’s Wright/SAT, 2ME, blood culture, and bone marrow culture. In some cases, aspiration of intraocular fluids followed by culture and serology and biopsy may be necessary.[2][5][6] In our patient with uveitis, the confirmed diagnosis was serologically performed through positive results foranti-brucella antibodies while rejecting the other common causes of uveitis.

Patients with posterior uveitis or panuveitis have a poor prognosis.[9] The major complications of ophthalmic brucellosis include reduced vision and blindness of various degrees, cataract, glaucoma, maculopathy, viteral alteration, retinal neovascular membrane, tractional retinal detachment, and phthisis bulbi.[2][3][4][8][10][11][12]

Standard treatment with administration of rifampin and doxycycline and in case of eye involvement with local and systemic corticosteroide for 2-4 weeks leads to considerable improvement.[4][5][12]

Uveitis is the most common form of eye involvement and therefore, patients with uveitis in endemic areas for brucellosis should also be investigated for brucellosis. Since the patients with no ophthalmic symptoms still may have ophthalmic involvement, it is recommended that in endemic areas patients with systemic brucellosis should be thoroughly examined for ophthalmic involvement.

## References

[1] Hatipoglu CA, Yetkin A, Ertem GT, Tulek N (2004). Unusual clinical presentation of brucellosis. Scand J Infect Dis.

[2] Rolando I, Olarte L, Vilchez G, Lluncor M, Otero L, Paris M, Carrillo C, Gotuzzo I (2008). Ocular Manifestations Associated with Brucellosis: A 26-year Experience in Peru. Clin Infect Dis.

[3] Puig Solanes M, Heatley J, Arenas F, Guerrero Ibrra G (1953). Ocular Complications in brucellosis. Am J ophthalmol.

[4] Rabinowitz R, Schneck M, Levy J, Lifshitz T (2005). Bilateral Multifocal Choroiditis with Serous Retinal Detachment in a Patient with Brucella Infection: Case Report and Review of the literature. Arch Ophthalmol.

[5] Moutray TN, Williams MA, Best RM, McGinnity GF (2007). Brucellosis: a forgotten cause of uveitis?. Asian J Ophthalmol.

[6] Rolando I, Vilchez G, Olarte L, Lluncor M, Carrillo C, Paris M, Guerra H, Gottuzzo E (2009). Brucellar uveitis: intraocular fluids and biopsy studies. Int J Infect Dis.

[7] al-Kaff AS (1995). Ocular brucellosis. Int ophthalmol Clin.

[8] Güngür K, Bekir NA, Namiduru M (2002). Ocular Complications associated with brucellosis in an endemic area. Eur J Ophthalmol.

[9] Rolando I, Tobaru L, Hinostroza S, Guerra L, Carbone A, Carrillo C, Gotuzzo E (1987). Clinical manifestations of brucellar uveitis. Ophthalmil Practice.

[10] Meek B, Speijer D, de Jong PT, de Smet MD, Peek R (2003). The ocular humoral immune response in health and disease. Prog Retin Eye Res.

[11] Maini R, O`Sullivan J, Reddy A, Watson S, Edelsten C (2004). The risk of complications of uveitis in a district hospital cohort. Br J Ophthalmol.

[12] Lashay AR, Manaviat MR, Azimi R, Riazi M (2006). Ocular Complications of Brucellsis: a case Report. Ophthalmic and Vision Research Journal.

